# A Linear-Scaling Integral-Direct Explicitly Correlated Second-Order Møller–Plesset Approach

**DOI:** 10.1021/acs.jctc.5c02184

**Published:** 2026-03-03

**Authors:** Mihály Kállay, Péter R. Nagy, Bence Ladóczki, Dávid Mester

**Affiliations:** † Department of Physical Chemistry and Materials Science, Faculty of Chemical Technology and Biotechnology, 415618 Budapest University of Technology and Economics, Műegyetem rkp. 3, Budapest H-1111, Hungary; ‡ HUN-REN−BME Quantum Chemistry Research Group, Műegyetem rkp. 3, Budapest H-1111, Hungary; § MTA−BME Lendület Quantum Chemistry Research Group, Műegyetem rkp. 3, Budapest H-1111, Hungary

## Abstract

We present an integral-direct, iteration-free, linear-scaling, local explicitly correlated second-order Møller–Plesset (MP2-F12) approach, extending our previous local MP2 method [*J. Chem. Theory Comput.* **2016**, 12, 4897]. The correlation contributions for individual electron pairs are computed within domains defined by the corresponding localized orbitals, while the correlation energies of spatially distant electron pairs are determined via multipole expansions. All the various types of integrals are computed and transformed directly, thereby precluding the need for integral storage and yielding asymptotically constant memory as well as negligible disk I/O demand. Another competitive advantage is the implementation of the 2B MP2-F12 ansatz, the most complete one so far with local approximations, enabling excellent basis set convergence even with double-ζ basis sets. Our validation studies indicate that the approach recovers at least 99.9% of the canonical MP2-F12 correlation energy and yields reaction energies with a mean error of less than 1 kJ/mol. With respect to the complete basis set limit of MP2, the error of our local approach is just slightly larger than that of canonical MP2-F12. With the new local MP2-F12 approach, we were able to compute the correlation energy for a small protein containing 644 atoms, which is the largest system ever considered in an explicitly correlated calculation.

## Graphical abstract

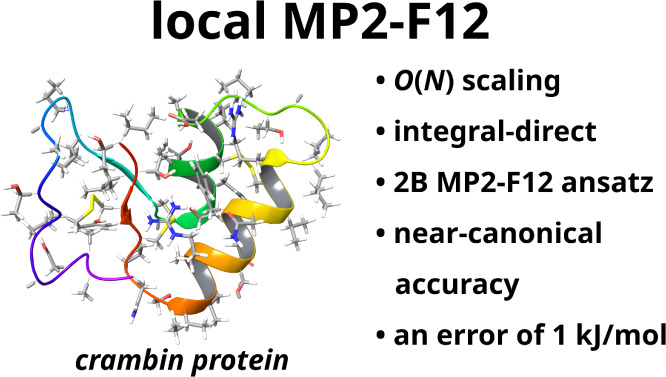

## 1 Introduction

Second-order Møller–Plesset (MP) perturbation theory (MP2)^1^ is one of the most popular approaches in computational chemistry. Even though advanced density functional theory (DFT) approximations can provide similar accuracy at lower computational expenses, it is widely used for the treatment of weak interactions and also in combination with DFT approaches in double hybrid functionals. The relatively high cost of MP2 calculations is a consequence of its fifth-power scaling with the system size, which is aggravated by its strong dependence on the quality of the atomic orbital (AO) basis set size. Over the years, numerous approaches have been proposed to mitigate these problems.

The scaling of MP2 can be reduced even to linear invoking the various AO-based and local correlation MP2 approximations. Methods in the first category utilize the sparsity of integrals and wave function parameters expressed in the AO basis and remove energy denominators via Laplace transform (LT).^2,3^ AO-based techniques were pioneered by Ayala and Scuseria^4^ and later perfectionized by Ochsenfeld and coworkers,^5–8^ and several other related approaches have also been put forward.^9,10^ Local MP2 (LMP2) approaches rely on the sparsity of the various quantities expanded in the molecular orbital (MO) basis. Usually, the canonical occupied Hartree–Fock (HF) MOs are localized, and the resulting localized MOs (LMOs) are applied together with some local virtual orbitals such as projected AOs (PAOs) to make use of the locality of electron correlation. The development of local correlation methods was initiated by Pulay^11^ and Kapuy^12^ and further continued by Werner and coworkers.^13–16^ More recently, Neese and coworkers combined the pair natural orbital (PNO) technique with local correlation,^17,18^ which inspired several groups to develop LMP2 approaches based on PNOs or related orbitals.^19–24^ The aforementioned LMP2 methods solve the corresponding equations simultaneously for the entire system as the amplitude equations needed for MP2 are coupled in the basis of LMOs. In another major class of local correlation approaches the system is divided into small fragments, and the correlation contributions of the individual fragments are evaluated separately. Notable examples for the fragmentation-based LMP2 methods are the various incremental methods initiated by Stoll,^25,26^ the divide-and-conquer approach of Kobayashi and Nakai,^27^ the divide-expand-consolidate (DEC) scheme of Jørgensen et al.,^28,29^ and the cluster-in-molecule (CIM) approach of Li and coworkers.^30–34^

Our LMP2 method^35–37^ combines advantageous properties of the above coupled and fragmentation methods and can be put in a third, uncoupled category, as discussed in ref 38. Our LMP2 approach utilizes advanced versions of the pair- and domain approximation introduced by Pulay and also used by PNO-based LMP2 methods. Unlike these coupled or direct methods, using LT-based techniques, our LMP2 amplitude equations are uncoupled. This makes the use of natural orbitals unnecessary at the MP2 level. In these aspects, our LMP2 scheme bears more resemblance to the CIM and incremental techniques. However, our LMP2 approach avoids techniques representative of fragmentation methods (fragmentation to subsystems, bond cutting, capping, etc.) or any other real-space-based or system-dependent cutoffs. These advantageous properties enabled our LMP2 calculations for systems of more than 2000 atoms with a triple-ζ basis set^39^ and over 1000 atoms close to the complete basis set (CBS) limit.^40^

One way to overcome the slow basis set convergence of MP2 is through explicitly correlated methods.^41–43^ These approaches speed up calculations by using wave functions that explicitly contain the distances between electrons, therefore requiring much smaller basis sets. The theory began in the mid-1980s with Kutzelnigg and Klopper,^41,44,45^ who developed the first explicitly correlated MP2 using a simple linear distance factor termed R12.^45^ The theory has advanced significantly through several critical improvements. The linear R12 factor was replaced by an exponential factor, denoted as F12,^46^ which provides higher accuracy, and the fixed amplitude approach was developed to simplify the equations without sacrificing precision.^47^ The essential approach used to calculate the matrix elements, the resolution of the identity (RI) approximation, was made more accurate and stable using auxiliary basis sets instead of the MO basis.^48^ An alternative variant of this approach, the complementary auxiliary basis set (CABS) scheme, was also developed, where the union of the MO and auxiliary basis sets is utilized in the RI.^49^ Computational efficiency was increased by applying the density fitting (DF) technique for integral evaluation^50^ and by developing alternative matrix element formulations, which also optimize convergence toward the CBS limit.^51^ These innovations have resulted in MP2-F12 theory becoming a highly robust and essential tool in modern computational chemistry.^52–54^

LMP2 theory was first combined with explicit correlation by Werner and Manby.^55^ This approach utilized PAOs for the virtual space, the linear R12 correlation factors, and the DF approximation. Subsequently, this method was considerably improved by introducing the exponential correlation factor^56^ and a local ansatz for the strong-orthogonality projector,^57^ which is required to keep explicitly correlated terms orthogonal to the conventional excitation space. A periodic variant of the approach was also developed by Usvyat, which facilitates the use of explicitly correlated MP2 theory for solids.^58^ Recently, the AO-based MP2 technique was also extended to explicit correlation by Ochsenfeld et al.,^59^ who proposed an efficient algorithm for the evaluation of the most expensive exchange-type term of MP2-F12 theory utilizing numerical quadratures.

LMP2-F12 theory was also combined with the PNO technology. The first such method was presented by Tew et al., who demonstrated the advantages of PNOs for the compression of both the virtual and the complementary space without reducing the scaling of MP2-F12.^60^ The reduced-scaling extension of this approach was also published more recently.^61^ The first reduced-scaling PNO-based LMP2-F12 approach was developed by Valeev and coworkers,^62^ and linear scaling was also achieved soon thereafter utilizing the domain based local PNO (DLPNO) methodology of Neese et al.^63^ The resulting DLPNO-MP2-F12 method was significantly more expensive than the parent DLPNO-MP2 approach, and apart from one-dimensional model systems, it was employed for medium-sized systems of at most 150 atoms, primarily with double-ζ basis sets. The PNO technique was also adapted by Werner and coworkers, who reported an efficient, highly parallelized PNO-LMP2-F12 approach, which enables applications for more extended systems.^64^ In a recent application, this method was applied to a molecular complex of 260 atoms,^65^ which was probably the largest system previously considered in an explicitly correlated calculation. However, this LMP2-F12 approach, unlike the aforementioned ones, uses the ansatz 3*A, which introduces less accurate approximations.^52^ For instance, the coupling of conventional and explicitly correlated configurations is completely neglected, which alone can lead to errors of more than 2 and 1 kcal/mol in computed reaction energies with double- and triple-ζ basis sets, respectively.^66^

Concerning fragmentation-based LMP2 approximations, a couple of them was also extended to explicit correlation. Friedrich and coworkers implemented an automated incremental scheme for F12 methods including MP2-F12, but their approach was applicable to relatively small systems with a couple of dozens of atoms.^67,68^ Kristensen et al. developed a massively parallel LMP2-F12 approach in the DEC context.^69^ Even though their algorithm is linear scaling, it was only tested for relatively small molecules of fewer than 50 atoms. The other notable fragmentation-based LMP2-F12 implementation is the one presented by Li and coworkers.^70^ They combined the CIM approach with explicitly correlated methods and also incorporated the DLPNO approximation to accelerate the calculation of fragment correlation contributions. The largest real-life test system considered in their study was a molecular complex of 133 atoms with a double-ζ basis set, while triple-ζ calculations were performed for smaller systems of less than 100 atoms.

Here, we discuss the explicitly correlated extension of our linear scaling LMP2 method formulated in refs 35 and 36, which was gradually improved and extended in refs 39,40 and 37. We use a more rigorous MP2-F12 ansatz than in the previous linear scaling MP2-F12 approaches reported in the literature and introduce only local approximations. Our main design goal is to preserve the accuracy of the parent MP2-F12 method with respect to CBS-limit MP2. Parallel to that, our intention is to also retain the favorable features of our LMP2 implementation, such as the integral-direct and iteration-free algorithm as well as the use of LT to reduce the scaling of domain MP2-F12 calculations, and thereby to enable calculations for larger systems.

## 2 Theory

First, we briefly review MP2-F12 theory and our LMP2 approach. We only focus on the features that are indispensable for the understanding of the following discussion. More details can be found in the literature^47,51,54^ and in our previous publications.^35–37,71–73^ Subsequently, the explicitly correlated extension of our LMP2 model is presented.

### 2.1 Explicitly Correlated MP2

#### 2.1.1 MP2-F12 Ansatz

Assuming a closed-shell HF reference function with spin-restricted orbitals, the conventional MP2 correlation energy can be expressed as

1 $$E_{c}^{\mathrm{MP}2}=\underset{abij}{\sum }\left[2\left(ai|bj\right)-\left(aj|bi\right)\right]t_{ij}^{ab}$$

where (*ai*|*bj*) denotes four-center electron repulsion integrals (ERI) in Mulliken’s convention, whereas indices *i*, *j*, ... (*a*, *b*, ...) stand for occupied (virtual) HF orbitals. Indices *p*, *q*, ... will refer to general orbitals in the HF MO basis. In the case of a canonical HF reference, first-order amplitudes $t_{ij}^{ab}$ can be evaluated as

2 $$t_{ij}^{ab}=\frac{\left(ai|bj\right)}{D_{ij}^{ab}}$$

where $D_{ij}^{ab}$ is an energy denominator, $D_{ij}^{ab}=\varepsilon_{i}+\varepsilon_{j}-\varepsilon_{a}-\varepsilon_{b}$ with ε_*p*_ as the corresponding orbital energies.

In MP2-F12 theory, the wave function explicitly incorporates the interelectronic distance *r*_12_ through specific pair functions, the F12 correlation factors,

3 $$f_{12}=-\frac{1}{\gamma }e^{-\gamma r_{12}}$$

where γ is an exponent. The three- and four-electron integrals appearing due to the presence of these pair functions are approximated by inserting the RI at particular places. For that purpose, a formally complete MO basis is needed. In the CABS approach, the HF MO basis is augmented with complementary virtual orbitals. In practice, an AO-like auxiliary basis is utilized, whose elements are orthogonalized to the HF MO space and then orthonormalized to each other. The orbitals derived from this representation of the complementary virtual space will be indexed by *a*′, *b*′, ..., while *p*′, *q*′, ... will label arbitrary orbitals in the formally infinite basis.

The MP2-F12 correlation energy can be expressed as a sum of the classical MP2 correlation energy and the contribution of the explicitly correlated terms, $E_{c}^{F12}$ :

4 $$E_{c}^{\mathrm{MP}2-F12}=E_{c}^{\mathrm{MP}2}+E_{c}^{F12}$$

For the latter, we apply ansatz 2B as well as the F + K commutator and the fixed amplitude approximations.^47,51,54^ To avoid any confusion, we note that we adhere to the nomenclature of ref 54, and the approximation used here was termed ansatz C in ref 51. Then, $E_{c}^{F12}$ can be split up into four contributions as

5 $$E_{c}^{F12}=\underset{i\leq j}{\sum }\frac{1}{1+\delta_{ij}}\left(B_{ij}-X_{ij}+C_{ij}+V_{ij}\right)$$

where *B*, *X*, *C*, and *V* are intermediates depending on Fock matrix elements and integrals of operators 1/*r*_12_, *f*_12_, $f_{12}^{2}$ , *f*_12_/*r*_12_, and (∇̂_1_ *f*_12_)^2^ with ∇̂_1_ as the del operator with respect to the coordinates of electron one. For example, intermediate *C*, which describes the coupling of conventional and explicitly correlated configurations, reads as

6 $$C_{ij}=\frac{1}{16}\underset{ab}{\sum }\left[40\left(ai|bj\right)-8\left(aj|bi\right)+7R_{ij}^{ab}+R_{ji}^{ab}\right]{\;r}_{ij}^{ab}$$

where, in a canonical HF basis,

7 $$r_{ij}^{ab}=\frac{R_{ij}^{ab}}{D_{ij}^{ab}}$$

Here, intermediate $R_{ij}^{ab}$ is obtained by transforming the two-electron integrals of operator *f*_12_, (*a*′*i*|*f*_12_|*bj*), with the Fock matrix:

8 $$R_{ij}^{ab}=\underset{a^{\prime }}{\sum }f_{aa^{\prime }}\left(a^{\prime }i|f_{12}|bj\right)+\underset{b^{\prime }}{\sum }f_{bb^{\prime }}\left(ai|f_{12}|b^{\prime }j\right)$$

where $f_{aa^{\prime }}$ denotes Fock matrix elements. Our working equations for the other intermediates can be found in ref 71.

Usually, the MP2-F12 correlation energy is a rather accurate approximation to the CBS-limit of MP2 correlation energy, and the basis set error of the HF energy is considerably larger. To reduce the latter, an efficient solution is to compute the CABS singles correction,^74,75^ which is a simple, second-order correction to the HF energy.

#### 2.1.2 Density Fitting

In practical realizations of MP2 and MP2-F12 theories, most modern implementations exploit the DF approximation. In this approach, another AO-like auxiliary basis is utilized, and orbital products are expanded in this basis. ERIs can be recast as the product of three- and two-center Coulomb integrals, (*pq*|*P*) and *V*_*P*,*Q*_ = (*P*|*Q*), respectively, as

9 $$\left(pq|rs\right)\approx \underset{P,Q}{\sum }\left(pq|P\right)(V^{-1}{)}_{P,Q}\left(Q|rs\right)=\underset{P}{\sum }J_{pq,P}J_{rs,P}$$

10 $$J_{pq,P}=\underset{Q}{\sum }\left(pq|Q\right)L_{Q,P}$$

where *P*, *Q*, ... run over the functions of the fitting basis. Matrix **L** is formally obtained by some appropriate decomposition of matrix **V** ^–1^. In our implementation, the Cholesky-decomposition (CD) of matrix **V** is computed, and matrix **J** is evaluated by solving the corresponding linear system of equations. For the integrals of the four other operators, the so-called robust fitting formulas are employed.^50,54,76^ For instance, for the integrals of operator *f*_12_, these expressions read as

11 $$\left(pq|f_{12}|rs\right)\approx \underset{P}{\sum }\left(J_{pq,P}K_{rs,P}+K_{pq,P}J_{rs,P}\right)$$

where

12 $$K_{pq,P}=\underset{Q}{\sum }\left(pq|f_{12}|Q\right)L_{Q,P}-\frac{1}{2}\underset{Q,R,S}{\sum }J_{pq,Q}L_{R,Q}\left(R|f_{12}|S\right)L_{S,P}$$

and (*pq*|*f*_12_|*Q*) and (*R*|*f*_12_|*S*) stand for the three- and two-center integrals of operator *f*_12_.

### 2.2 Local MP2 Approach

#### 2.2.1 Local MP2 Ansatz

In our LMP2 approach, the MP2 correlation energy is evaluated as

13 $$E_{c}^{\mathrm{LMP}2}=\underset{i}{\sum }\delta E_{i}^{\mathrm{MP}2}\left(E_{i}\right)+{\underset{ij}{\sum }}^{\prime }\delta E_{ij}^{\mathrm{MP}2}\left(P_{ij}\right)$$

where, in this equation and hereafter, *i*, *j*, ... refer to LMOs. $\delta E_{i}^{\mathrm{MP}2}\left(E_{i}\right)$ is the contribution of LMO *i* to the correlation energy computed in the local domain $E_{i}$ , the so-called extended domain (ED) of *i*:

14 $$\delta E_{i}^{\mathrm{MP}2}\left(E_{i}\right)=\underset{abj\in E_{i}}{\sum }\left[2\left(ai|bj\right)-\left(aj|bi\right)\right]t_{ij}^{ab}$$

where the summation runs over the orbitals of the ED, and *a*, *b*, ... hereafter stand for orthonormalized PAOs. LMO *i* will be referred to as the central MO (CMO) of $E_{i}$ . The second summation in eq 13 is restricted to the *i* – *j* pairs of LMOs that are not included in any ED. $\delta E_{ij}^{\mathrm{MP}2}\left(P_{ij}\right)$ is an approximation to the MP2 pair correlation energy of orbitals *i* and *j* computed within pair domain $P_{ij}$ , which is the union of the smaller orbital-specific domains of orbitals *i* and *j*, the so-called primary domains (PDs). In addition to the domain approximation, at the evaluation of $\delta E_{ij}^{\mathrm{MP}2}\left(P_{ij}\right)$ , the same-spin component of the pair energy is also neglected, and the remaining opposite-spin contribution is calculated invoking the multipole approximation for the required ERIs as detailed in ref 36.

#### 2.2.2 Domain Construction

After solving the HF self-consistent field (SCF) equations, our LMP2 algorithm starts with the localization of orbitals, for which the Boys method^77^ is used by default, and the construction of PAOs. Subsequently, the various domains are assembled. First, applying a modified Boughton–Pulay (BP) algorithm,^78^ to each LMO and PAO, the atoms are assigned on which these orbitals are localized.^36^ In short, this algorithm selects an atom list for each MO so that the overlap of the MO and its projection onto the AOs residing on the atoms of the list will be larger than a predefined threshold *T*. For the latter, which is also referred to as the BP completeness criterion, different default values are used for the various types of orbitals and domains. For the construction of PDs, BP atom lists with thresholds *T*_PDo_ = 0.999 and *T*_PDv_ = 0.98 are determined for the LMOs and PAOs, respectively. Two further BP atom lists are generated for the occupied orbitals of the EDs with default values of *T*_EDo_ = 0.9999 and *T*_o_ = 0.985. The atom lists determined by the above thresholds will be referred to as BPPDo, BPPDv, BPEDo, and BPo domains, respectively.

Next, the various atom and orbital domains are assembled. The MO basis of a PD incorporates the given LMO and the PAOs derived from the AOs of the atoms of the BPPDo domain of the LMO. The atom list of the PD is the union of the BPPDo domain and the BPPDv atom lists of the aforementioned PAOs. The latter are truncated to the atoms of the PD, and the PAOs are orthonormalized to each other and to the LMO. Finally, the PAOs are canonicalized, that is, the Fock-matrix is diagonalized in their space. After assembling the PDs, the approximate pair energies $\delta E_{ij}^{\mathrm{MP}2}\left(P_{ij}\right)$ are computed, where pair domains $P_{ij}$ are simply the union of the corresponding PDs. Pairs for which $\delta E_{ij}^{\mathrm{MP}2}\left(P_{ij}\right)\geq \varepsilon_{w}$ are considered strong pairs, where the default value of threshold ε_w_ is 10^–5^ *E*_h_, enabled by the use of improved pair correlation energy estimates.^36^ The occupied MO basis of ED $E_{i}$ contains LMO *i*, that is, the CMO of the ED, and all other LMOs that form a strong pair with *i*. The ED’s atom list is the union of the BPEDo domains of these occupied orbitals, and the AO list of the ED consists of the AOs centered on these atoms. To select the PAOs for the ED, a PAO-center domain (PCD) is also constructed by merging the BPo atom lists of the occupied LMOs of the ED. PAOs derived from the AOs of these atoms are added to the MO basis of the ED. If warranted by integral estimates, additional AOs and PAOs can be added to the ED.^38,39^ The occupied orbitals are truncated to their own BPEDo domains, while the PAOs to the entire ED. The occupied and virtual spaces of the ED are separately orthonormalized and canonicalized. The resulting pseudocanonical orbitals will be labeled by *ĩ*, *j̃*, ... and *ã*, *b̃*, ..., respectively. Finally, a local density fitting domain is assembled incorporating the DF auxiliary functions that are used within the ED to approximate the two-electron ERIs. For this purpose, the auxiliary functions residing on the atoms of the PCD are applied, which includes only a subset of the entire atom list of the ED.

#### 2.2.3 Integral Evaluation

The calculation of the correlation energy contribution of the CMO requires the evaluation of the integrals in the MO basis of the ED. In the first step, which is the rate-determining one, (μ*i*|*P*)-type integrals are computed in an integral direct manner. Here, μ is an AO basis function of the ED, *P* is an auxiliary function from the local fitting domain, and *i* is an LMO of the ED truncated to its BPEDo domain. At the calculation and transformation of the AO integrals, it is utilized that *i* spans over only a limited number of atoms, and a sophisticated prescreening technique is also applied. In the second step, the remaining AO index is transformed to the pseudocanonical virtual basis to arrive at integrals (*ãi*|*P*). Finally, index *i* is transformed to the pseudocanonical occupied basis, that is, (*ãĩ*|*P*)-type integrals are computed. The latter two steps are performed by Basic Linear Algebra Subprograms (BLAS)-accelerated matrix multiplications and are relatively fast.

#### 2.2.4 Evaluation of Energy Contributions

The naive calculation of the correlation energy contribution, eq 14, would scale as the fifth-power of the ED’s size. This is because, in this case, one would assemble (*ãĩ*|*b̃j̃*)-type integrals, which is a fifth-power scaling operation, evaluate the first-order amplitudes according to the canonical expression, eq 2, transform index *ĩ* of the resulting $t_{ĩ\mathrm{j̃}}^{ã\mathrm{b̃}}$ amplitudes to *i*, and contract them with the corresponding integrals. To avoid this, we elaborated an alternative procedure based on CD or LT to eliminate the energy denominators, which reduces the scaling to fourth-order.^35,36^ In our algorithm, the first-order amplitudes with one of the occupied indices being the CMO are evaluated as

15 $$t_{i\mathrm{j̃}}^{ã\mathrm{b̃}}=-\underset{\omega }{\sum }\underset{P}{\sum }J_{ãi,P}^{\omega }J_{\mathrm{b̃}\mathrm{j̃},P}^{\omega }$$

where ω runs over the Cholesky-vectors or the Laplace integration quadrature points. Integrals $J_{\mathrm{b̃}\mathrm{j̃},P}^{\omega }$ are defined by

16 $$J_{\mathrm{b̃}\mathrm{j̃},P}^{\omega }=J_{\mathrm{b̃}\mathrm{j̃},P}{\left(e_{\omega }\right)}_{\mathrm{b̃}\mathrm{j̃}}$$

where ${\left(e_{\omega }\right)}_{\mathrm{b̃}\mathrm{j̃}}$ stands for an element of the Cholesky-vector or, when LT is employed,

17 $$(e_{\omega }{)}_{\mathrm{b̃}\mathrm{j̃}}=\sqrt{w_{\omega }}{\;e}^{-(\varepsilon_{\mathrm{b̃}}-\varepsilon_{\mathrm{j̃}})t_{\omega }}$$

with *t*_ω_ and *w*_ω_ as the quadrature points and weights, respectively. Integrals $J_{ãi,P}^{\omega }$ are computed from $J_{ãĩ,P}^{\omega }$ by transforming its index *ĩ* to the CMO *i*. Finally, the MP2 correlation contribution of LMO *i* is computed as

18 $$\delta E_{i}^{\mathrm{MP}2}\left(E_{i}\right)=\underset{ã\mathrm{b̃}\mathrm{j̃}\in E_{i}}{\sum }\left[2\left(ãi|\mathrm{b̃}\mathrm{j̃}\right)-\left(ã\mathrm{j̃}|\mathrm{b̃}i\right)\right]{\;t}_{i\mathrm{j̃}}^{ã\mathrm{b̃}}$$

### 2.3 Local MP2-F12 Approach

Here, we discuss step by step what modifications are necessary to our LMP2 approach outlined in Section 2.2 to incorporate explicit correlation.

#### 2.3.1 Local MP2-F12 Ansatz

In principle, the $E_{c}^{F12}$ term of eq 4 could be decomposed similar to the MP2 correlation energy, eq 13, as the sum of orbital and pair energy contributions. However, $E_{c}^{F12}$ accounts for the short-term part of the correlation energy, and we can suppose that the contribution of explicitly correlated terms to the pair energies of distant pairs—the ones that are included in the summation in the last term of eq 13—is negligible. Consequently, we only consider orbital contributions and define our LMP2-F12 correlation energy as

19 $$E_{c}^{\mathrm{LMP}2-F12}=\underset{i}{\sum }\delta E_{i}^{\mathrm{MP}2}\left(E_{i}\right)+{\underset{ij}{\sum }}^{\prime }\delta E_{ij}^{\mathrm{MP}2}\left(P_{ij}\right)+\underset{i}{\sum }\delta E_{i}^{F12}\left(E_{i}\right)$$

where $\delta E_{i}^{F12}\left(E_{i}\right)$ is the contribution of LMO *i* to $E_{c}^{F12}$ evaluated in ED $E_{i}$ :

20 $$\delta E_{i}^{F12}\left(E_{i}\right)=\frac{1}{2}\underset{j\in E_{i}}{\sum }\left(B_{ij}-X_{ij}+C_{ij}+V_{ij}\right)$$

Our LMP2-F12 total energy also includes the CABS singles correction. The evaluation of the latter, just as the computation of the $\delta E_{i}^{F12}\left(E_{i}\right)$ contributions, necessitates the calculation of the Fock-matrix in the joint AO plus CABS basis. This is a time-consuming task, but as discussed in ref 79, its scaling can be reduced even to linear utilizing local DF and domain approximations.^36,80–82^

#### 2.3.2 Domain Construction

We found that the domain structure developed for LMP2 is also a good starting point for LMP2-F12, but, of course, a few modifications are needed. First, an RI basis is required for the calculation of the $\delta E_{i}^{F12}\left(E_{i}\right)$ contributions. In line with our LMP2 model, we use a local RI approximation, that is, a local RI basis is constructed in each ED separately. Thus, as the core orbitals are part of the RI basis, the corresponding ones are added to the EDs. For that reason, the core orbitals are localized, separately from the correlated occupied MOs, and the BPEDo atom list are also generated for the localized core MOs, following our approach in ref 79. To avoid the unnecessary extension of the EDs, a core orbital is added to an ED should its entire BPEDo atom list be covered by the atoms of that ED. In this way, the core MOs of the atoms that are located farther from the CMO may be excluded from the ED, but our experience shows that this has no direct bearing on the accuracy of the RI approximation. Besides the core MO’s BPEDo list, additional BP atom lists, termed as BPtrf, are also compiled for the correlated LMOs with a default threshold of *T*_trf_ = 0.99999. These BPtrf domains will be useful at the integral transformations as discussed below, and the determination of the default *T*_trf_ will be presented in Section 3.2.

Second, the infrastructure for the local CABS must be established. For the local RI approximation, the most plausible choice is to include the CABS functions of all the atoms of the ED. However, we found that even a subset of these functions still yields accurate results. Thus, we introduced a new domain, hereafter referred to as the CABS domain, that includes the atoms and the corresponding CABS functions considered in the RI. To avoid the unnecessary proliferation of domains, only existing ones were tested as the CABS domain: the entire ED itself, the PCD, and the BPEDo and BPo domains of the CMO, which are subsets of each other with decreasing sizes around the CMO. As demonstrated in Section 3.2, BPEDo is sufficient for all purposes, and accordingly, it is used as default for the CABS domain.

To generate a local complementary virtual space, the AO basis of the ED is projected out from the CABS functions of the CABS domain. The resulting functions are Löwdin symmetric orthogonalized dropping the eigenvectors of their overlap matrix with eigenvalues lower than 10^–7^. Typically, the MO space of the ED, that is, the space spanned by the core and correlated LMOs and orthonormalized PAOs of the ED, is smaller than the ED’s AO basis. Consequently, it may happen that important functions are missing from the RI, which is composed of the functions of the MO space and the complementary virtual space constructed from the CABS functions. To fill the gap between the MO and complementary virtual spaces, the latter is augmented with further functions. The functions of the MO space are projected out of the AOs belonging to the atoms of the CABS domain. These projected AOs are also Löwdin orthogonalized with a threshold 10^–4^ on the eigenvalues of the overlap matrix, and the resulting functions are added to the complementary virtual space. This extended space together with the MO space of the ED are used as RI basis within the ED. The functions of the complementary virtual space are, of course, located close to the CMO, therefore, the RI approximation may be less accurate for the contributions of the LMOs that are farther from the CMO. Nevertheless, these contributions and consequently the error caused are expected to be moderate.

#### 2.3.3 Integral Evaluation

To evaluate the energy contributions in the ED, the following types of three-center integrals are required: (*p*′*i*|*P*), (*p*′*i*|*f*_12_|*P*), $\left(p^{\prime }i|f_{12}^{2}|P\right)$ , (*ji*|*f*_12_/*r*_12_|*P*), (*ji*|(∇̂_1_ *f*_12_)^2^|*P*), where *p*′ labels a function of the RI space of the ED, *i* and *j* are correlated LMOs, and *P* is an auxiliary function of the PCD. The calculation of these integrals follows a three-step route similar to the LMP2 case.

First, integrals (μ′*i*|*P*), (μ′*i*|*f*_12_|*P*), $\left(\mu^{\prime }i|f_{12}^{2}|P\right)$ , (μ*i*|*f*_12_/*r*_12_|*P*), and (μ*i*|(∇̂_1_ *f*_12_)^2^|*P*) are computed with an integral-direct algorithm employing intensive prescreening. Here, μ denotes the AO basis functions of the ED, while μ′ is a function of the AO plus CABS basis of the ED, that is, the space that includes all the AOs of the ED and the CABS functions of the CABS domain. An important difference with respect to our LMP2 approach is that here, the LMOs are truncated to a larger domain. As revealed in Section 3.2, the use of the BPEDo domains would cause unacceptably large error in the explicitly correlated terms, especially at the calculation of interaction energies. Consequently, we consider here the larger BPtrf domains, but to avoid the unnecessary enlargement of the ED, their intersection with the ED is used at the integral transformation. In this manner, the truncation of LMOs close to the edge of the ED will be less accurate than 0.99999 (but still better than 0.9999), however, their contribution and the error introduced are anticipated to be small.

In the second step, the μ′ or μ index of the half-transformed integrals is transformed, respectively, to the RI or the original LMO basis of the ED. Finally, the index *i* of the integrals is transformed back to the original LMO basis of the ED. The integral evaluation concludes with the formation of intermediates **J** and **K** defined by eqs 10 and 12, respectively. The second half transformation and the subsequent operations are again performed as simple matrix multiplications and are therefore efficient. This is in contrast to the first half transformation, which is the rate-determining step of our entire LMP2-F12 algorithm.

#### 2.3.4 Evaluation of Energy Contributions

First, the contributions of intermediates *B*, *X*, and *V* to $\delta E_{i}^{F12}\left(E_{i}\right)$ are computed. It is easy to see that these contributions are invariant to the unitary transformation of occupied MOs, and thus, they can be evaluated directly in the LMO basis of the ED. Hence, this can simply be implemented by straightforward modifications of the canonical MP2-F12 code. Note that one of the indices of these intermediates is fixed, i.e., the CMO of the ED, therefore, their evaluation scales as the fourth-power of the size of the ED. After calculating these contributions, the integrals of operators $f_{12}^{2}$ , *f*_12_/*r*_12_, and (∇̂_1_ *f*_12_)^2^ are dropped as they are not needed any more, whereas the occupied indices of intermediates **J** and **K** are transformed to the pseudocanonical occupied basis of the ED.

The evaluation of the contribution of intermediate *C* is less trivial since $r_{ij}^{ab}$ of eq 7 is not orbital-invariant but assumes canonical MOs. In consequence, similar to the MP2 correlation contribution, the brute-force implementation of this term would also result in an algorithm that scales as the fifth-power of the ED’s size, with a significantly larger prefactor to make matters worse. However, eliminating the energy denominator, we can again design a fourth-power scaling algorithm. To that end, let us define the

21 $${\mathrm{J̅}}_{ãĩ,P}=\underset{a^{\prime }}{\sum }f_{ãa^{\prime }}J_{a^{\prime }ĩ,P}$$

and

22 $${\mathrm{K̅}}_{ãĩ,P}=\underset{a^{\prime }}{\sum }f_{ãa^{\prime }}K_{a^{\prime }ĩ,P}$$

Fock-transformed fitting coefficients. Analogously to $J_{\mathrm{b̃}\mathrm{j̃},P}^{\omega }$ , see eq 16, we can also introduce intermediates, ${\mathrm{J̅}}_{ãĩ,P}^{\omega }$ , $K_{\mathrm{b̃}\mathrm{j̃},P}^{\omega }$ , and ${\mathrm{K̅}}_{ãĩ,P}^{\omega }$ . Then, $r_{ij}^{ab}$ can be expressed in the ED of CMO *i* as

23 $$r_{i\mathrm{j̃}}^{ã\mathrm{b̃}}=-\underset{\omega }{\sum }\underset{P}{\sum }\left({\mathrm{J̅}}_{ãi,P}^{\omega }K_{\mathrm{b̃}\mathrm{j̃},P}^{\omega }+{\mathrm{K̅}}_{ãi,P}^{\omega }J_{\mathrm{b̃}\mathrm{j̃},P}^{\omega }+J_{ãi,P}^{\omega }{\mathrm{K̅}}_{\mathrm{b̃}\mathrm{j̃},P}^{\omega }+K_{ãi,P}^{\omega }{\mathrm{J̅}}_{\mathrm{b̃}\mathrm{j̃},P}^{\omega }\right)$$

With these quantities, utilizing its unitary invariance, intermediate *C* can be computed as

24 $$C_{i\mathrm{j̃}}=\frac{1}{16}\underset{ã\mathrm{b̃}\in E_{i}}{\sum }\left[40\left(ãi|\mathrm{b̃}\mathrm{j̃}\right)-8\left(ã\mathrm{j̃}|\mathrm{b̃}i\right)+7R_{i\mathrm{j̃}}^{ã\mathrm{b̃}}+R_{\mathrm{j̃}i}^{ã\mathrm{b̃}}\right]{\;r}_{i\mathrm{j̃}}^{ã\mathrm{b̃}}$$

where

25 $$R_{i\mathrm{j̃}}^{ã\mathrm{b̃}}=\underset{P}{\sum }\left({\mathrm{J̅}}_{ãi,P}K_{\mathrm{b̃}\mathrm{j̃},P}+{\mathrm{K̅}}_{ãi,P}J_{\mathrm{b̃}\mathrm{j̃},P}+J_{ãi,P}{\mathrm{K̅}}_{\mathrm{b̃}\mathrm{j̃},P}+K_{ãi,P}{\mathrm{J̅}}_{\mathrm{b̃}\mathrm{j̃},P}\right)$$

In the above equations, ${\mathrm{J̅}}_{ãi,P}^{\omega }$ , ${\mathrm{K̅}}_{ãi,P}^{\omega }$ , and $K_{ãi,P}^{\omega }$ are obtained from ${\mathrm{J̅}}_{ãĩ,P}^{\omega }$ , ${\mathrm{K̅}}_{ãĩ,P}^{\omega }$ , and $K_{ãĩ,P}^{\omega }$ , respectively, by transforming their *ĩ* index to the CMO, and similar relations hold for the corresponding intermediates without index ω. Finally, the contribution of the coupling term to the domain energy is evaluated as

26 $$\delta E_{i}^{F12}\left(E_{i}\right)\leftarrow \frac{1}{2}\underset{\mathrm{j̃}\in E_{i}}{\sum }C_{i\mathrm{j̃}}$$

We have found that the same number of Cholesky-vectors/quadrature points, typically 6 to 8, as for the MP2 correlation contribution are sufficient also for the evaluation of the coupling term.

#### 2.3.5 Scaling of the Algorithm

As all the operations within the ED are asymptotically independent of the system size, the calculation of the MP2-F12 correlation energy is linear scaling. Clearly, our algorithm still contains steps with scaling higher than linear, although these typically consume a small fraction of the total computation time. These steps include the PAO construction and the calculation of the $\delta E_{ij}^{\mathrm{MP}2}\left(P_{ij}\right)$ pair energies, which scale as the third and second power of the system size, respectively. In addition, all the major steps preceding the correlation energy calculation, such as the HF-SCF iteration, the calculation of the Fock matrix in the formally complete basis, the evaluation of the CABS singles correction, and the orbital localization are cubic scaling. Among them, only the first two ones require computation times comparable to that of the correlation energy calculation and may act as a bottleneck.

As to the memory consumption, the size of the arrays in an ED scales at most cubically with the size of the ED, and thus, it is asymptotically independent of the system size. Consequently, the scaling of the memory requirement for the ED calculations is sublinear. On the other hand, there are particular arrays kept in memory whose size exhibits linear or even quadratic scaling. These include, for example, the basis set information, particular mappings, PAO coefficients, and the Fock matrix in the joint AO plus CABS basis of the entire molecule. In practice, we have found that only the latter can become a bottleneck, but only for very large molecules; the memory requirement is usually determined by the three-index arrays of the EDs. The asymptotically constant scaling of the disk I/O beyond the SCF and localization steps is also a favorable property of our LMP2-F12 algorithm.

## 3 Benchmark Calculations

### 3.1 Computational Details

The LMP2-F12 approach presented in this paper has been implemented in the mrcc quantum chemical program and will be available in the next release of the package.^83,84^ mrcc is used in all the calculations reported herein.

To evaluate the accuracy of the method, test calculations were carried out for various test sets. The basic settings were determined utilizing the test set of Neese, Wennmohs, and Hansen (NWH)^17^ and the S66 set of Rezáč and coworkers.^85^ The former includes 23 reactions of organic molecules of up to 36 atoms, while the latter is a collection of weak interaction energies of 66 dimers of at most 34 atoms. To further validate our findings, our method was also tested for benchmark sets containing bigger systems: the ISOL test set of Grimme et al.^86^ and the L7 set of Hobza and coworkers.^87^ The ISOL set is composed of 24 reaction energies of 48 organic molecules including up to 81 atoms, while L7 is a compilation of binding energies of molecular complexes of at most 112 atoms. To analyze the performance of our LMP2-F12 ansatz, we employed a standard set of statistical error metrics. These include the mean absolute error (MAE), root-mean-square (RMS) deviation, and the maximum absolute error (MAX). Here, we only present the error statistics of the computed energy differences. The errors for the correlation energies can be found in the Supporting Information (SI) together with the breakdown of the errors to various correlation energy contributions.

Timing benchmarks were performed for even larger systems in the 128- to 644-atom range. These include two DNA fragments of two and four adenine-thymine base pairs abbreviated, respectively, by DNA_2_ and DNA_4_,^5^ the vancomycin molecule,^88^ and a small protein, crambin.^88^ For these systems, at the HF-SCF and Fock matrix computations, local fitting domains were applied as described in ref 36. Thresholds of 1, 10^–3^, and 1 *E*_h_ were used, respectively, for the SCF iterations, the final SCF iteration, and the computation of the Fock matrix in the formally complete basis. The timing benchmarks were carried out on a 18-core Intel Xeon Gold 6254 CPU running at 3.1 GHz allocating at most 1 TB of main memory to the calculations.

In the explicitly correlated calculations, the correlation-consistent polarized valence *X*-tuple-ζ F12 basis sets (cc-pV*X*Z-F12, *X* = D, T, and Q) of Peterson et al.^89^ were employed. For brevity, the cc-pV*X*Z-F12 basis sets will be abbreviated as *X*Z. As recommended, the exponent γ of the *f*_12_ correlation factor was set to 0.9, 1.0, and 1.1 for *X* = D, T, and Q, respectively.^89^ For the CABS, the corresponding cc-pV*X*Z-F12-OPTRI basis of Yousaf and Peterson^90,91^ were applied. In all the MP2-F12 calculations, the ERIs were approximated by DF both for the HF and the correlation energy. For this purpose, the aug-cc-pV­(*X* + 1)­Z-RI-JK^92^ and aug-cc-pV*X*Z-RI^93^ auxiliary bases of Weigend were selected, respectively. For the large-scale applications presented in Section 3.3, the aug-cc-pVTZ-RI-JK auxiliary basis was employed at the HF level with the cc-pVTZ-F12 basis set. We note that otherwise, we usually use the more complete aug-cc-pwCV­(*X* + 1)­Z-RI weighted core-valence auxiliary basis set^94^ for the correlation energy calculation by default as recommended by the developers of explicitly correlated theories.^54^ These fitting basis sets are desirable to accurately approximate the integrals of core orbitals and high angular momentum functions appearing in RI bases. To test how large the error caused by the smaller auxiliary bases is, the canonical MP2-F12 energies were computed for the NWH, S66, and ISOL test suites with both types of fitting basis. The results, which can be found in the SI, show that the errors are negligible for the TZ and QZ basis sets, while with DZ, they are comparable to the error of our local approximations and almost an order of magnitude smaller than the basis-set error of DZ MP2-F12 correlation energies. These findings suggest that it is reasonable to apply the aug-cc-pV*X*Z-RI auxiliary bases within our LMP2-F12 approach.

For the NWH, S66, and ISOL test sets, reference MP2 correlation energies were computed with Dunning’s augmented correlation consistent *X*-tuple-ζ basis sets, aug-cc-pV*X*Z (*X* = Q, 5, 6).^95–98^ These calculations also employed the DF approximation together with the corresponding aug-cc-pV*X*Z-RI auxiliary bases.^92,93^ In the case of the NWH and ISOL test sets, the diffuse functions of the hydrogen atoms were removed from aug-cc-pV6Z-RI to avoid linear dependencies in the basis set. CBS-limit MP2 correlation energies were estimated by the two-point formula of ref 99 using the aug-cc-pV5Z and aug-cc-pV6Z results. For reactions 1 and 4 of the ISOL test set, we were unable to perform the aug-cc-pV6Z calculations. For these reactions, the aug-cc-pVQZ and aug-cc-pV5Z values were considered at the extrapolation. For further reference, the relevant error statistics for canonical MP2-F12 are compiled in Table 1.

**Table 1:** Errors (in kJ/mol) of Canonical MP2-F12 Correlation Contributions to Energy Differences with Respect to CBS MP2 Reference Values for the NWH, S66, and ISOL Test Sets with Various Basis Sets

		Basis set		
Test set	Error measure	cc-pVDZ-F12	cc-pVTZ-F12	cc-pVQZ-F12
NWH	MAE	0.7	0.4	0.3
	RMS	1.0	0.6	0.5
	MAX	3.1	1.5	1.2
S66	MAE	0.2	0.3	0.2
	RMS	0.2	0.3	0.3
	MAX	0.5	0.9	0.8
ISOL	MAE	0.8	0.6	-
	RMS	1.0	0.9	-
	MAX	2.2	2.8	-

In all the calculations, CD were employed to remove the energy denominators in the domains. The number of Cholesky-vectors was automatically determined to reduce the diagonal elements of the residual matrix to less than 10^–4^ a.u.^100^

### 3.2 Determination of Default Parameters

The basic parameters of our LMP2-F12 approach were optimized by test calculations performed for the NWH and S66 test sets. Our design goal was to keep the relative error of correlation energies below 0.1% and the MAE (MAX) below 1 kJ/mol (1 kcal/mol) for energy differences with respect to the corresponding MP2-F12 results evaluated in the same basis set. In addition, we endeavored not to worsen the basis set incompleteness error of the parent MP2-F12 method. Thus, compared to genuine MP2-F12, we aimed at an increase of at most 1 kJ/mol in any error measure with respect to CBS-limit MP2.

First, supposing that the default settings of our LMP2 model given in Section 2.2 are also appropriate for LMP2-F12, we determined the size of the CABS domain. As mentioned in Section 2.3, four options were tested for that purpose: the ED, PCD, BPEDo, and the BPo domains. The results are summarized in Table 2, where the error statistics are shown with respect to both the canonical MP2-F12 results obtained in the same basis set and the CBS-limit MP2 references.

**Table 2:** Errors (in kJ/mol) of LMP2-F12 Correlation Contributions to Energy Differences with Respect to Canonical MP2-F12 (CBS MP2 Reference) Values for the NWH and S66 Test Sets with Various Basis Sets as a Function of the CABS Domain Size

			Domain			
Test set	Basis set	Error measure	ED	PCD	BPEDo	BPo
NWH	cc-pVDZ-F12	MAE	0.5 (0.9)	0.5 (0.9)	0.5 (0.9)	1.1 (1.3)
		RMS	0.6 (1.0)	0.6 (1.0)	0.6 (1.0)	1.4 (1.6)
		MAX	1.4 (2.0)	1.4 (2.0)	1.5 (1.9)	3.3 (4.4)
	cc-pVTZ-F12	MAE	0.5 (0.5)	0.5 (0.5)	0.5 (0.5)	0.5 (0.6)
		RMS	0.7 (0.7)	0.7 (0.7)	0.7 (0.7)	0.7 (0.8)
		MAX	1.9 (2.5)	1.9 (2.5)	1.8 (2.5)	1.7 (2.7)
	cc-pVQZ-F12	MAE	0.3 (0.4)	0.3 (0.4)	0.3 (0.4)	0.3 (0.4)
		RMS	0.4 (0.5)	0.4 (0.5)	0.4 (0.5)	0.4 (0.5)
		MAX	1.1 (1.0)	1.1 (1.0)	1.1 (1.0)	1.1 (1.0)
S66	cc-pVDZ-F12	MAE	1.9 (1.7)	1.9 (1.7)	1.9 (1.7)	1.6 (1.4)
		RMS	2.1 (2.0)	2.1 (2.0)	2.1 (1.9)	1.8 (1.7)
		MAX	4.6 (4.6)	4.6 (4.6)	4.5 (4.5)	4.3 (4.3)
	cc-pVTZ-F12	MAE	1.8 (1.5)	1.8 (1.5)	1.7 (1.5)	1.7 (1.4)
		RMS	1.9 (1.7)	1.9 (1.7)	1.9 (1.7)	1.9 (1.7)
		MAX	4.1 (3.8)	4.1 (3.8)	4.1 (3.8)	4.1 (3.8)
	cc-pVQZ-F12	MAE	1.7 (1.5)	1.7 (1.5)	1.7 (1.5)	1.7 (1.5)
		RMS	2.0 (1.8)	2.0 (1.8)	2.0 (1.8)	2.0 (1.8)
		MAX	4.6 (4.4)	4.6 (4.4)	4.6 (4.4)	4.6 (4.4)

With the DZ basis set, the reaction energy errors for the NWH compilation practically do not change even if the CABS domain is shrank to BPEDo but suddenly grow when using BPo. In the latter case, the errors with respect to both same-basis MP2-F12 and CBS-limit MP2 are unacceptable. Obviously, this is caused by the steep increase of the errors of correlation energies (see SI). They are not larger than 0.05% with the larger domains, but the maximum error goes up to 0.20% when switching to BPo. The errors for the S66 interaction energies are also independent of the CABS domain size up to BPEDo, but interestingly, they drop when BPo is applied. However, it seems to be a fortunate error compensation because the correlation energy errors increase considerably. Concerning the larger basis sets, the errors of energy differences are virtually independent of the choice of the CABS domain, and similar holds for the correlation energies. Accordingly, for the TZ and QZ basis sets even BPo can safely be used as the CABS domain. Nevertheless, to be on the conservative side, we recommend BPEDo as the default CABS domain.

The results in Table 2 also show that, while the reaction energies are sufficiently accurate with the BPEDo CABS domain, the errors of the interaction energies do not comply with our design goals irrespective of the CABS domain and the basis set. Analyzing this issue, we concluded that this is caused by the truncation of the LMOs to speed up the integral transformation. As explained in Section 2.2, LMOs truncated to the BPEDo domain are employed in our original LMP2 algorithm at the integral transformation. While it is sufficient for MP2 correlation energies, our experience showed that the integrals computed in this way are not accurate enough for the evaluation of the F12 contributions. To obviate this, as outlined in Section 2.3, we introduced the larger BPtrf domains, whose size is controlled by the *T*_trf_ BP completeness threshold. To set its default value, further test calculations were carried out for the NWH and S66 test sets with the recommended BPEDo CABS domain and *T*_trf_ thresholds that were tighter than *T*_EDo_. The results are compiled in Table 3.

**Table 3:** Errors (in kJ/mol) of LMP2-F12 Correlation Contributions to Energy Differences with Respect to Canonical MP2-F12 (CBS MP2 Reference) Values for the NWH and S66 Test Sets with Various Basis Sets as a Function of the *T*_trf_ Truncation Parameter

			*T*_trf_		
Test set	Basis set	Error measure	0.99995	0.99999	0.999995
NWH	cc-pVDZ-F12	MAE	0.3 (0.9)	0.1 (0.8)	0.1 (0.7)
		RMS	0.4 (1.2)	0.2 (1.1)	0.2 (1.1)
		MAX	0.8 (3.4)	0.6 (3.7)	0.6 (3.4)
	cc-pVTZ-F12	MAE	0.2 (0.5)	0.1 (0.5)	0.1 (0.5)
		RMS	0.3 (0.7)	0.1 (0.7)	0.1 (0.7)
		MAX	0.7 (1.8)	0.4 (1.8)	0.4 (1.9)
	cc-pVQZ-F12	MAE	0.3 (0.4)	0.1 (0.4)	0.1 (0.3)
		RMS	0.4 (0.5)	0.2 (0.5)	0.2 (0.5)
		MAX	1.0 (1.3)	0.5 (1.6)	0.5 (1.6)
S66	cc-pVDZ-F12	MAE	1.0 (0.8)	0.2 (0.2)	0.2 (0.2)
		RMS	1.2 (1.0)	0.3 (0.3)	0.3 (0.3)
		MAX	2.8 (2.8)	1.1 (1.0)	0.8 (0.7)
	cc-pVTZ-F12	MAE	0.9 (0.6)	0.3 (0.2)	0.2 (0.2)
		RMS	1.0 (0.8)	0.4 (0.3)	0.3 (0.3)
		MAX	1.9 (1.7)	1.2 (0.9)	1.1 (0.8)
	cc-pVQZ-F12	MAE	0.9 (0.7)	0.3 (0.2)	0.2 (0.2)
		RMS	1.0 (0.8)	0.4 (0.3)	0.3 (0.3)
		MAX	2.0 (1.8)	1.4 (1.2)	1.2 (1.0)

The results show that the reaction energies are quite accurate even with the loosest threshold, which is not surprising as they were satisfactory already with *T*_trf_ = *T*_EDo_ (see Table 2). The errors of reaction energies practically converge with *T*_trf_ = 0.99999. The dependence of interaction energies on the *T*_trf_ threshold is more pronounced. Our design criteria are met in all the cases if the errors relative to the same-basis MP2-F12 results are considered, though particular error measures are on the borderline. However, the MAXs with respect to CBS-limit MP2 are still too large in the DZ and QZ basis sets (cf. Table 1). The errors are reduced typically by a factor of 2 to 3 when going to *T*_trf_ = 0.99999 and acceptable in all respects. The improvement is marginal when *T*_trf_ is tightened to 0.999995. With *T*_trf_ = 0.99999, the quality of the correlation energies is also more than acceptable as their relative error is 0.02% in the worst case. Taking into account the above observations, we select *T*_trf_ = 0.99999 as our default threshold for the size of the BPtrf domain.

To verify the accuracy of our LMP2-F12 approach with its default settings for larger systems, test calculations were performed for the ISOL and L7 compilations. The corresponding error statistics are presented in Table 4.

**Table 4:** Errors (in kJ/mol) of LMP2-F12 Correlation Contributions to Energy Differences with Respect to Canonical MP2-F12 (CBS MP2 Reference) Values for the ISOL and L7 Test Sets with Various Basis Sets

Test set	Basis set	Error measure		
ISOL	cc-pVDZ-F12	MAE	0.5	(0.8)
		RMS	0.9	(1.1)
		MAX	3.6	(2.3)
	cc-pVTZ-F12	MAE	0.5	(1.0)
		RMS	0.9	(1.6)
		MAX	3.5	(5.1)
L7	cc-pVDZ-F12	MAE	0.5	-
		RMS	0.6	-
		MAX	0.9	-

Regarding reaction energies, we can conclude that the errors are considerably bigger than those for the NWH test set (see the *T*_trf_ = 0.99999 column in Table 3). Of course, we should keep in mind that the ISOL benchmark set includes more complicated reactions with more bonds breaking and forming. The average errors with respect to the parent MP2-F12 method are absolutely acceptable, while the maximum error is unexpectedly large though still tolerable. The culprit is reaction 8, which was also challenging for other LMP2-F12 approaches.^101^ Here, the electronic structures of the reactant and the product are not only especially complicated but also significantly differ from each other. With one exception, the error of the other reaction energies is smaller than 1 kJ/mol. As to the error with respect to CBS-limit MP2 (cf. Table 1), we observe that the error measures hardly increase compared to genuine MP2-F12 in the DZ basis set. With TZ, the increments in the MAE and RMS are still reasonable, but the MAX increases by 2.1 kJ/mol. This is again caused by reaction 8, where the errors of the local approximations in our LMP2-F12 method and the intrinsic basis-set error of MP2-F12 are additive. Apart from this reaction, the increment in the error is also larger than 1 kJ/mol for reaction 4, which is similarly complicated. For other reactions, the accuracy of the LMP2-F12 approach is satisfactory.

The interaction energies of the L7 test set are very accurate. Even though these complexes are larger and exhibit more complicated electronic structure than those in the S66 compilation, and their binding energies are roughly an order of magnitude larger, the error measures hardly change compared to S66. We also note that the correlation energies of the larger systems in the ISOL and L7 benchmark sets are still satisfactorily precise: we did not find any system where the relative error would have been greater than 0.07%.

### 3.3 Representative Applications

To gain some insight into the computational demands of our LMP2-F12 method and to demonstrate its potential for large molecular systems, benchmark calculations were carried out for the DNA_2_, vancomycin, DNA_4_, and crambin molecules, which feature 128, 176, 260, and 644 atoms, respectively. In all the calculations, the default settings were used at the evaluation of the LMP2-F12 correlation energies. The results obtained with the cc-pVDZ-F12 and cc-pVTZ-F12 basis sets are presented in Tables 5 and 6, respectively.

**Table 5:** Wall-Clock Times in Minutes for LMP2-F12 Computations on Large Molecules with the cc-pVDZ-F12 Basis Set

	DNA_2_	Vancomycin	DNA_4_	Crambin
No. of atoms	128	176	260	644
No. of AO basis functions	2766	3723	5712	12717
No. of CABS basis functions	6160	8316	12672	28556
HF	163	419	1247	16386
Fock matrix^a^	36	112	462	3243
CABS singles correction	0.5	1.5	4	39
Localization	0.1	1	1	9
LMP2-F12 correlation energy	1411	3436	11358	57445
Total LMP2-F12	1611	3970	13072	77122

a Evaluation of the Fock matrix in the formally complete basis.

**Table 6:** Wall-Clock Times in Minutes for LMP2-F12 Computations on Large Molecules with the cc-pVTZ-F12 Basis Set

	DNA_2_	Vancomycin	DNA_4_
No. of AO basis functions	4982	6721	10264
No. of CABS basis functions	7832	10650	16032
HF^a^	255	697	2353
Fock matrix^b^	133	229	675
CABS singles correction	2	4	7
Localization	0.4	1	2
LMP2-F12 correlation energy	4130	8781	24453
Total LMP2-F12	4521	9712	27490

a Restarted from converged cc-pVDZ-F12 densities.

b Evaluation of the Fock matrix in the formally complete basis.

As it can be seen, the computation time is dominated by the evaluation of the correlation energy in both basis sets. Except for the largest example, crambin, the HF-SCF calculation is an order of magnitude less expensive than the correlation energy. This is in contrast to our conventional LMP2 method, where the situation is reversed, especially for bigger systems.^36^ Of course, we should keep in mind that even without local approximations, MP2-F12 is at least an order of magnitude more expensive than conventional MP2 even if their scaling properties are identical. For crambin, the HF calculation still consumes less computer time, but the evaluation of the correlation energy takes only three times longer. The computation of the Fock matrix in the joint AO and CABS basis also demands considerable resources. For the systems studied, the computer time spent for this operation amounts to 20 to 50% of that for the HF-SCF. The costs of the CABS correction and the localization are negligible.

While the LMP2-F12 timings are notable, the cost is compensated by high accuracy and excellent scaling to very large systems. Notice also that the above timings were measured on a single processor, but thanks to the independent domain computations and the integral-direct algorithm, the present LMP2-F12 method is expected to be parallelizable with high efficiency. Another convenient approach to speed up our LMP2-F12 model, which is also trivial to implement, is to combine it with conventional LMP2 in a multilevel framework. That is, the chemically important part can be treated at the LMP2-F12 level, while its surrounding with LMP2. Such embedding techniques were previously developed for our conventional local correlation methods^102^ and were also applied to explicitly correlated approaches by others.^103^

## 4 Conclusions

In this paper, an integral-direct, iteration-free, linear-scaling local MP2-F12 approach has been presented. Beyond these features, a key component of our method is the use of Cholesky decomposition or the Laplace transform to reduce the scaling associated with calculating correlation contributions within the local domains. An important advantage of our scheme over other linear-scaling LMP2-F12 methods is that it is based on the theoretically most complete MP2-F12 ansatz, which guarantees fast basis-set convergence and moderate basis-set error even with double-ζ basis sets. Other appealing attributes of our approach are the low-order scaling memory demand and the complete elimination of disk I/O by computing all the necessary integrals and intermediates on-the-fly. This design makes our approach attractive for computation on machines with relatively slow external storage, such as contemporary computer clusters. The combination of direct algorithms and the uncoupled local MP2 ansatz also inherently enables efficient parallelization, which will be the subject of future research. Our test calculations demonstrate that the new approach is highly accurate and allows for LMP2-F12 calculations for larger systems than previously possible. With this implementation, we successfully computed the LMP2-F12 correlation energy for a small protein containing 644 atoms—the largest system for which an explicitly correlated calculation has been performed to date.

The new approach itself is useful for the calculation of basis-set limit MP2 energies and energy differences for large molecules. More importantly, it can be employed to reduce the basis set error of other methods. One such application is the MP2-based basis-set correction for local coupled-cluster (CC) methods, where the difference of the LMP2-F12 and LMP2 energies is added to the CC energy.^104^ Another potential use case of our approach is to diminish the basis-set error of double hybrid DFT energies for large systems by calculating the second-order perturbation theory contribution with LMP2-F12.^105–107^ Finally, the new LMP2-F12 scheme will serve as the basis for linear-scaling, integral-direct, and disk I/O-free local explicitly correlated CC methods, which are currently developed in our laboratory.

## Supplementary Material



## Notes

Calculated correlation energy contributions; atomization; reaction; and interaction energies (XLSX)
